# Exploration of an XX/XY Sex Determination System and Development of PCR-Based Sex-specific Markers in *Procambarus clarkii* Based on Next-Generation Sequencing Data

**DOI:** 10.3389/fgene.2022.850983

**Published:** 2022-03-01

**Authors:** Yudong Shen, Qishuai Wang, Weimin Wang, Yanhe Li

**Affiliations:** Key Laboratory of Freshwater Animal Breeding, Ministry of Agriculture and Rural Affair/Engineering Research Center of Green Development for Conventional Aquatic Biological Industry in the Yangtze River Economic Belt, Ministry of Education, College of Fisheries, Huazhong Agricultural University, Wuhan, China

**Keywords:** sex determination system, sex-specific markers, *Procambarus clarkii*, next-generation sequencing, genetic sex

## Abstract

Many economic crustacean species have sex dimorphisms during their growth. Exploring the sex determination system and developing sex-specific molecular marker(s) are very helpful for carrying out sex control breeding, and next-generation sequencing has been used as an efficient way to explore them in recent years. In this study, first, the genetic sex determination system of *P. clarkii* was explored as an XX/XY system by analyzing the 2b-RAD sequencing data. Furthermore, DNA samples of male and female individuals from a *P. clarkii* family were pooled separately for whole-genome resequencing. Based on the data of whole-genome resequencing, the 9,163 male- and female-specific bias sites with higher feasibility were obtained based on the assumption of the XX/XY sex determination system, and four sites were selected to design the sex-specific marker primers. One efficient sex-specific marker was identified with a sex discrimination rate of 99.49% (195/196) when applied to five different geographical groups with 196 individuals. The results of this study would provide a foundation for the realization of *P. clarkii* sex control and could provide some reference for investigating the sex determination system and sex molecular marker(s) of other crustacean species based on next-generation sequencing data.

## Introduction

Large numbers of economic crustaceans, including prawn, shrimp, lobster, crayfish, and crab, have sex dimorphisms (Sun and Li, 2021). The red swamp crayfish, *Procambarus clarkii*, commonly known as ‘little lobster’ in China, which is native to the southern United States and northern Mexico (Huner, 1988), has recently become an important farmed aquatic economic species in China (Jin et al., 2019). There is sex dimorphism between the male and female morphology of *P. clarkii*. The meat content of female crayfish is higher than that of male crayfish in the same weight condition (Peng et al., 2021). So, all-female production could bring significant economic implications in aquaculture. Therefore, the realization of sex control of *P. clarkii* and unisexual culture of *P. clarkii* would have great market prospects. To carry out sex control breeding, it is necessary and helpful to know the sex determination system and develop sex-specific molecular markers. The sex-specific marker for identifying the genetic sex is an important tool to verify the success of sex reversal. In addition, the known sex determination system would be helpful for drafting its artificial breeding program. However, at present, the sex determination system of *P. clarkii* is not clear, and there are no sex-specific molecular markers in *P. clarkii*.

Generally, the genetic sex determination system of animals and plants is mainly either XX/XY or ZZ/ZW (Devlin and Nagahama, 2002). Prawns and shrimps, such as *Macrobrachium rosenbergii*, have an explicitly systematically defined ZZ/ZW sex determination system and have developed sex-specific molecular markers for obtaining sex reversal successfully (Malecha et al., 1992; Ventura et al., 2011; Levy et al., 2019), as well as *Penaeus monodon* (Staelens et al., 2008). For the crucifix crab (*Charybdis feriatus*), male-specific SNP markers were identified, and an XX/XY sex determination system was suggested based on the presence of male-specific SNP markers (Fang et al., 2020). With the decrease of sequencing cost, next-generation sequencing (NGS) has been used to explore or confirm the sex determination system of some species in recent years (Shi X. et al., 2018). In addition, SNP and InDel markers obtained by NGS are more efficient, economical, simple, and comprehensive than previous RAPD (Xia et al., 2011; Vale et al., 2014) and AFLP (Xu et al., 2013; Lai et al., 2014) methods. SNP and InDel markers reduced the possibility of failure in the development of trait association markers and provided new insights into the sex determination system (Shi X. et al., 2018; Fang et al., 2020).

In this study, we attempted to explore the sex determination system of *P. clarkii* and develop the sex-specific molecular marker(s) through NGS, which would be very helpful in enriching the basic theory of heredity in *P. clarkii* and provide a foundation for realization of *P. clarkii* sex control. This study could also provide some references for using NGS data to discover the sex determination system and sex molecular markers in other crustacean species.

## Materials and Methods

### Analysis of 2b-RAD Sequencing Data

In order to explore the sex determination system of *P. clarkii*, the raw data (SRP135662; 98 males and 122 females; Yi et al., 2018) in our laboratory were used for preliminary exploration. The BsaXI enzyme used in 2b-RAD sequencing technology can specifically recognize the “ACNNNNNCTCC” base sequence in the genome and digest the DNA sequence with this characteristic. Since the sequencing reads were added with the sequencing adapter, the clean reads with chain specificity were finally obtained, which was about 27 bp in length. The specific sequence of the enzyme digestion site was obtained based on 2b-RAD sequencing data of each individual crayfish. By removing redundant sequences and using Linux commands (e.g., uniq) to obtain the intersection and difference sets of 2b-RAD sequences in different samples, female-specific reads and male-specific reads were obtained to explore the sex determination system of *P. clarkii*.

### The Sample Collection and DNA Sample Preparation

To confirm the assumption of the sex determination system of *P. clarkii* inferred above, the six male and five female *P. clarkii* individuals were collected from one family for whole-genome resequencing. The physiological sex of *P. clarkii* was detected by external morphology and gonads. After being frozen with anesthesia, the muscle tissue of *P. clarkii* was collected for DNA extraction. Total DNA was extracted using the method described by Li et al. (2011). The qualified DNA strands (1.8 < A260/A280 < 2.1, the agarose gel electrophoresis DNA strip was clear, no obvious dispersion, and the degradation of small fragments was weak) were pooled into two DNA samples (female and male) and used for whole-genome resequencing.

In addition, a total of 196 crayfish including 105 females and 91 males (Supplementary Table S1) from Chaohu, Gaoyou, Yangxin, Honghu, and Hanchuan were selected, and the individual DNAs were prepared by the method mentioned above to testify the efficiency of sex-specific molecular markers.

### Library Construction and Fragment Screening

The two pooled DNA samples were used to construct the resequencing library. The BGISEQ sequencing platform (Beijing Genomics Institute, Beijing, China) was used to carry out the sequencing in the way of pair-end sequencing (150bp). The sequencing data volume of each sample was about 120 Gb, and the sequencing coverage/depth of each re-sequenced data point was set about 30×. SOAPnuke (Chen et al., 2018) was used to handle the original data to obtain clean data. The filtering parameters adopted by SOAPnuke were “- N 0.01—L 20—Q 0.3—a0.25—cutadapter—Q 2—G—polyx50—minlen 150”.

The MEM (maximal exact match) module of BWA (Burrows-Wheeler aligner tool) (Li and Durbin, 2010) was used to match the clean data to the reference genome of *P. clarkii* (PRJNA727411; Xu et al., 2021). The SAM files were converted to BAM files using SAMtools (Li et al., 2009). Duplicate reads were marked through GATK4’s MarkDuplicates module (McKenna et al., 2010), and then the marked BAM files were indexed by SAMtools. Each sample was individually detected for variation by using GATK4’s Haplotype Caller to obtain the gvcf intermediate file which would be combined into a final document by the CombineGVCFs module of GATK4 (genome analysis toolkit 4). Finally, the GenotypeGVCFs module of GATK4 was used to obtain all the variation information. All SNP and InDel sites were filtered with the filtering condition of SNPs, that is, QD < 2.0 || MQ < 40.0 || FS > 60.0 || SOR >3.0 || MQRankSum < −12.5 || ReadPosRankSum < −8.0, and the filtering condition of InDel is QD < 2.0 || FS > 200.0 || SOR >10.0 || MQRankSum < −12.5 || ReadPosRankSum < −8.0.

Based on the SNP and InDel sites obtained in the previous step, sex-linked SNP and InDel sites were further screened with more stringent parameters. The screening conditions are as follows: The sequencing coverage depth of each sequencing library at each site is greater than 30 ×; the female is homozygous and the male is heterozygous with the allele frequency close to 1:1.

### Primer Designing for PCR-Based Sex-Specific Molecular Marker and Experimental Verification

Primers are designed based on the region containing the InDel site, and one primer in a pair of primers overlapped the sequence region of the InDel site. The primers of Marker 1 and Marker 3 were designed based on the region of one InDel site, and the primers of Marker 6 and Marker 7 were based on another InDel site. All primers shown in Table 1 were designed by the Primer3 online website (http://primer3plus.com/cgi-bin/dev/primer3plus.cgi). The procedure of PCR was as follows: 94°C denaturation for 3 min, followed by 30 cycles with 94°C for 45 s, 56°C for 35 s, 72°C for 30 s, and then 72°C for 10 min for extension. The PCR products were run by electrophoresis with a concentration of 1.2% agarose gel (120 V, 15 min).

**TABLE 1:** Primers for developing PCR-based sex-specific molecular markers.

Primer name	Sequence	Product length	Location
Marker1_F	CAC​TTT​TGT​TCC​GGC​AAT​ACT​TCT	300–400 bp	Scaffold 38:18680471
Marker1_R	ATT​CAC​CTG​GAC​TTT​ATG​GGT​TGA		
Marker3_F	CTT​CCA​CTT​TTG​TTC​CGG​CAA​TAC	300–400 bp	Scaffold 38:18680471
Marker3_R	ACC​TGG​ACT​TTA​TGG​GTT​GAG​TTA		
Marker6_F	AGT​ACG​AGT​ATG​ACA​CTG​GTT​CAC	300–400 bp	Scaffold 38:17791823
Marker6_R	TAT​GGC​ATA​TAC​ATC​GTC​ACC​GTC		
Marker7_F	CGA​GTA​TGA​CAC​TGG​TTC​ACC​AAT	300–400 bp	Scaffold 38:17791823
Marker7_R	CAT​ATA​CAT​CGT​CAC​CGT​CCC​TG		

## Results

### Exploration of the Sex Determination System

**FIGURE 1:**
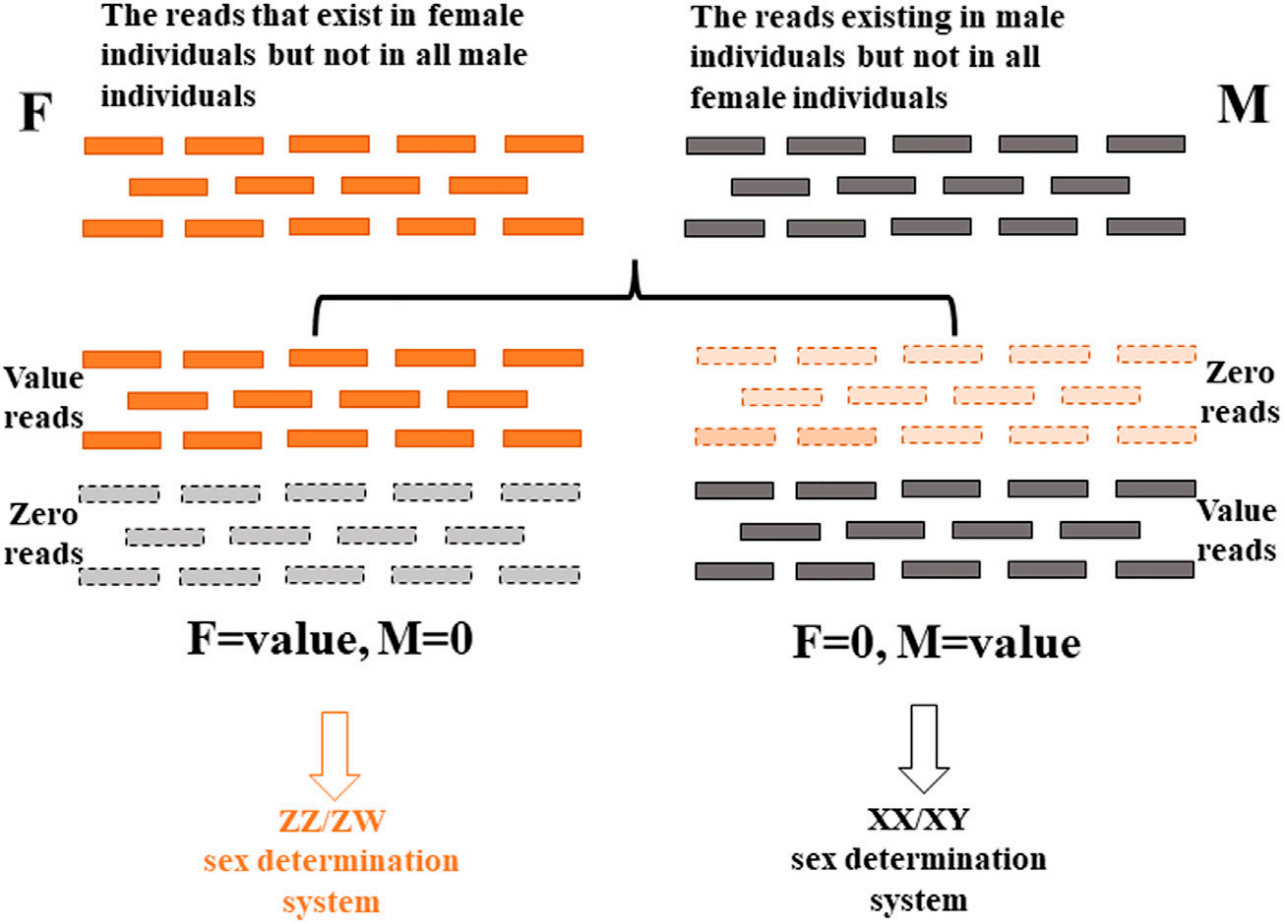
Schematic diagram of the overall research framework for exploring the sex determination system based on next-generation sequencing data.

The 2b-RAD sequencing raw data (SRP135662) in our laboratory were analyzed further and used to explore the sex determination system of *P. clarkii*. Theoretically, in the ZZ/ZW system, the reads that occurred in female samples and not in male samples might be from the W chromosome, while in the XX/XY system, the reads that occurred in male samples and not in female samples might be from the Y chromosome. Thus, after elimination of redundancy of clean reads of each sample, all clean reads were screened, and the reads existing in all male crayfish, but not in all female crayfish, were selected; the reads that exist in all female crayfish, but not in all male crayfish, were selected, each of which could reflect the XX/XY sex determination system and ZZ/ZW sex determination system, respectively. The overall research framework for the exploration of the gender determination system by analyzing the screened reads in this study is shown in Figure 1. The results showed that the number of reads existing in all male crayfish but not in all female crayfish was 160,609, which conforms to the hypothesis of the XX/XY sex determination system (Figure 1; Supplementary Table S2). Therefore, the sex determination system of *P. clarkii* was inferred as the XX/XY system.

### Statistics of Resequencing Data

The data of two resequencing samples (one for male and one for female) were obtained, and the resequencing data of each sample were about 120 Gb. The statistics results of the reads are shown in Table 2. The resequencing data have been deposited in NCBI GenBank under the accession number PRJNA797570. Based on the approximately 4 Gb genome size of *P. clarkii* (Shi L. et al., 2018; Xu et al., 2021), the sequencing coverage depth of each re-sequenced data point was about 30×.

**TABLE 2:** Statistics results of the reads of whole-genome resequencing.

Sample	Clean reads	Clean base	Read length (bp)	Q20 (%)	GC content (%)	Depth_mean	Coverage (%)
Males	854,329,136	128,149,370,400	150	95.60	41.97	57.81	97.52
Females	841,301,244	126,195,186,600	150	95.92	41.84	56.83	97.53

### Screening Results of SNP and InDel Sites With Male- and Female-Specific Bias

A total of 14,073,070 SNP sites and 3,495,983 InDel sites were obtained by GATK4. Among them, there were 1,625,684 SNP sites and 490,293 InDel sites (Supplementary Figure S1) under the assumption that the female group was homozygous and the male group was heterozygous.

Considering the randomness and preference of the distribution of sequencing data in the genome, the screening threshold was improved to ensure correlation strength between the sites and gender. The SNP and InDel sites were further screened and filtered by reserving the sequences; the sequencing coverage depth of each sequencing library at each site is greater than 30 × and the female is homozygous and the male is heterozygous with the allele frequency close to 1:1. In addition, a total of 9,163 SNP or InDel sites with male- and female-specific bias were obtained (Supplementary Table S3). The heterotopic points of gender difference are mainly concentrated on scaffold 38 (Supplementary Figure S2). According to the genome annotation information, all genes on scaffold 38 are functionally annotated (Supplementary Table S4).

### Experimental Verification of the Efficient Primers Designed

**FIGURE 2:**
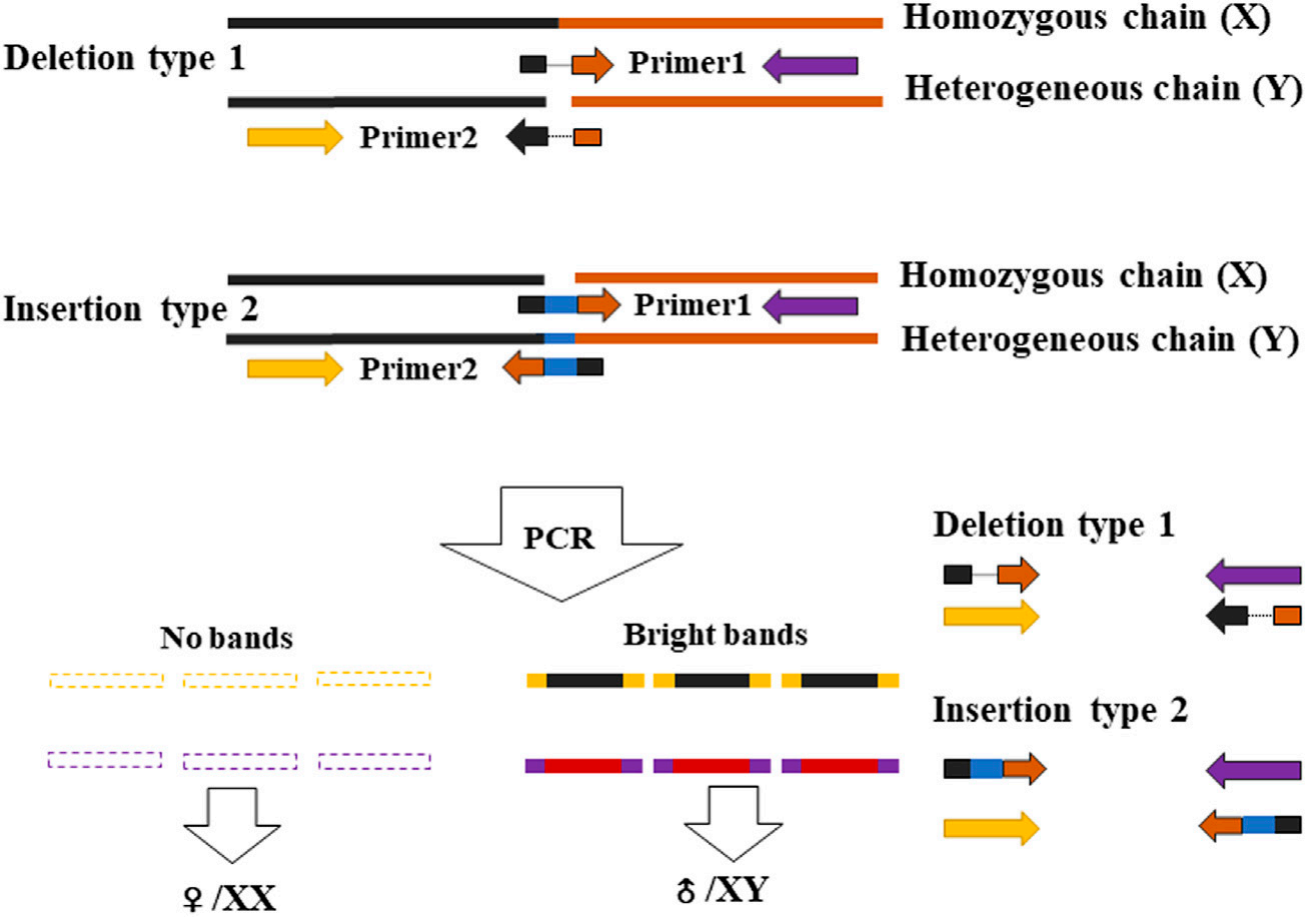
Schematic diagram of design principles of the sex-specific marker primers.

**FIGURE 3:**
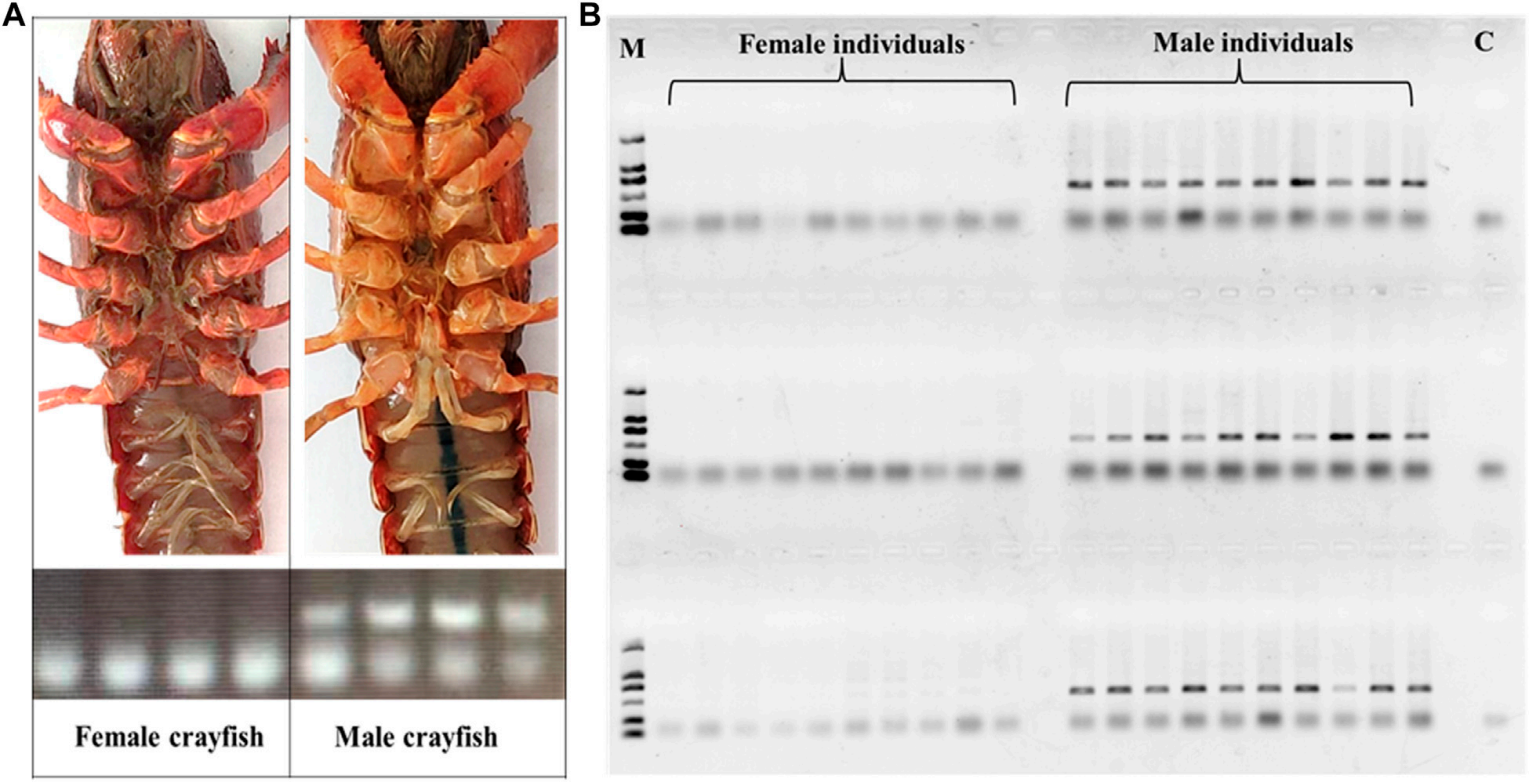
External form of *P. clarkii* sex and PCR detection. **(A)** External form of *P. clarkii* sex and PCR detection of female and male individuals. **(B)** PCR detection of 30 females and 30 males of *P. clarkii* by the primer pair Marker7_F/R. M, D 2000 DNA marker; C, negative control.

Based on the sites with male- and female-specific bias, the primers for sex-specific markers were designed and testified. Since one primer in a pair of primers overlaps the sequence region including InDel site(s) in the heterogenous chain (Figure 2), according to the assumption that the female group was homozygous and the male group was heterozygous, in theory, there is one specific amplified fragment (at 300–400 bp) which can appear in the PCR products of male *P. clarkii*, and there is no specific amplified fragment in the PCR products of female *P. clarkii* (Figure 3A). Four female and four male individuals were used to check the primers (showed in Table 1) first, and the difference between female and male crayfish is shown. All the four primer pairs we designed could distinguish between males and females. Then, more female and male crayfish with 60 individuals (30 female and 30 male crayfish) and 196 individuals from different geographic areas (details listed in Supplementary Table S1) were used sequentially to further test the primers. The results of agarose gel electrophoresis showed that all the PCR products of female crayfish did not contain the amplified fragment at 300–400 bp, while all the PCR products of male crayfish contained the specific amplified fragments at 300–400 bp using the primers of Marker7_F/R (Figure 3B) when 60 individuals were checked, and only one individual’s DNA was not amplified successfully when more crayfish (196 individuals) were checked by this primer pair. The sex discrimination rate was at least more than 99.49% (195/196). Of other primers (Marker1_F/R, Marker3_F/R, and Marker6_F/R), there were no specific amplified fragments appearing in the PCR products of all female *P. clarkii*, and there existed some geographical difference to some extent while detecting male crayfish (Supplementary Figure S3), in which some male individuals from the Anhui population could not be amplified successfully and did not contain the specific fragment. In addition, by comparing the sequence information of 50 kb up and down the gender molecular markers, it was found that there was no significant difference in sequencing depth between male and female samples (Supplementary Figure S4).

## Discussion

For species with sex dimorphism, it is helpful to obtain the characteristics of high stress resistance, rapid growth, and bright color under one certain gender by a unisexual breeding mode and to obtain higher yield or higher economic value (Lalrinsanga et al., 2012; Levy et al., 2017). It is necessary to realize their sex control breeding. The development of sex molecular markers and understanding of the sex determination mechanism are the bases for sex control breeding.

Generally, the genetic system of sex determination for a species could be uncovered through descent identification or karyotype analysis (Malecha et al., 1992; Ortega et al., 2017). Presently, high-throughput sequencing has been used as a rapid and efficient way to uncover the sex determination system of an organism (Pan et al., 2015; Ou et al., 2017; Shi X. et al., 2018; Gao et al., 2019; Yang et al., 2020). *P. clarkii* lacks visual heterogeneity chromosomes (Shi et al., 2019). In this study, through the sequencing data of 2b-RAD sequencing and whole-genome resequencing, we confirmed that the sex determination system of *P. clarkii* was the XX/XY system. From 2b-RAD sequencing data, the number of reads existing in all male crayfish but not in all female crayfish was 160,609, while the number of the reads that exist in all female crayfish but not in all male crayfish was zero, which conforms to the XX/XY sex determination system. Through the resequencing data, a total of 9,163 sites with male- and female-specific bias were obtained under the assumption of an XX/XY sex determination system in *P. clarkii*. Furthermore, based on the region containing the InDel site, four primer pairs were designed for PCR amplification, and almost all presented the same results that no amplified fragments appeared in the PCR products of female *P. clarkii*, and there were amplified fragments in some male individuals. Such results could also confirm that the sex determination system of *P. clarkii* was the XX/XY system. Further study would be focused on confirmation of the genetic sex determination system of *P. clarkii* by the method of descent identification. Of course, the sex of the crayfish might not be determined only by genetic factors and might also be affected by environmental factors, including temperature and density.

The efficiency of sex SNP and InDel markers developed at the genome level is higher. In this study, the sex SNP and InDel markers in *P. clarkii* were identified using resequencing. Of the four primer pairs, the primer pair of Marker7_F/R could distinguish between male and female crayfish from different geographical regions by the regular PCR method, and the success rate was 99.49% (195/196).

In conclusion, it has been confirmed that the genetic sex determination system of *P. clarkii* is the XX/XY system based on the NGS data. Four pairs of male-specific markers were designed, and one efficient sex-specific marker was identified with a sex discrimination rate of 99.49% when applied to different geographical groups.

## Data Availability

The original contributions presented in the study are included in the article/Supplementary Material; further inquiries can be directed to the corresponding author.
