# Supplementary material for: Exploration of an XX/XY Sex Determination System and Development of PCR-Based Sex-specific Markers in Procambarus clarkii Based on Next-Generation Sequencing Data
Source: Front Genet. 2022 Mar 1;13:850983. doi: 10.3389/fgene.2022.850983 (PMC8923706; doi:10.3389/fgene.2022.850983)
Supplement: Supplementary file 1 [file DataSheet1.ZIP › Supplemental materials/Figure S2.docx]

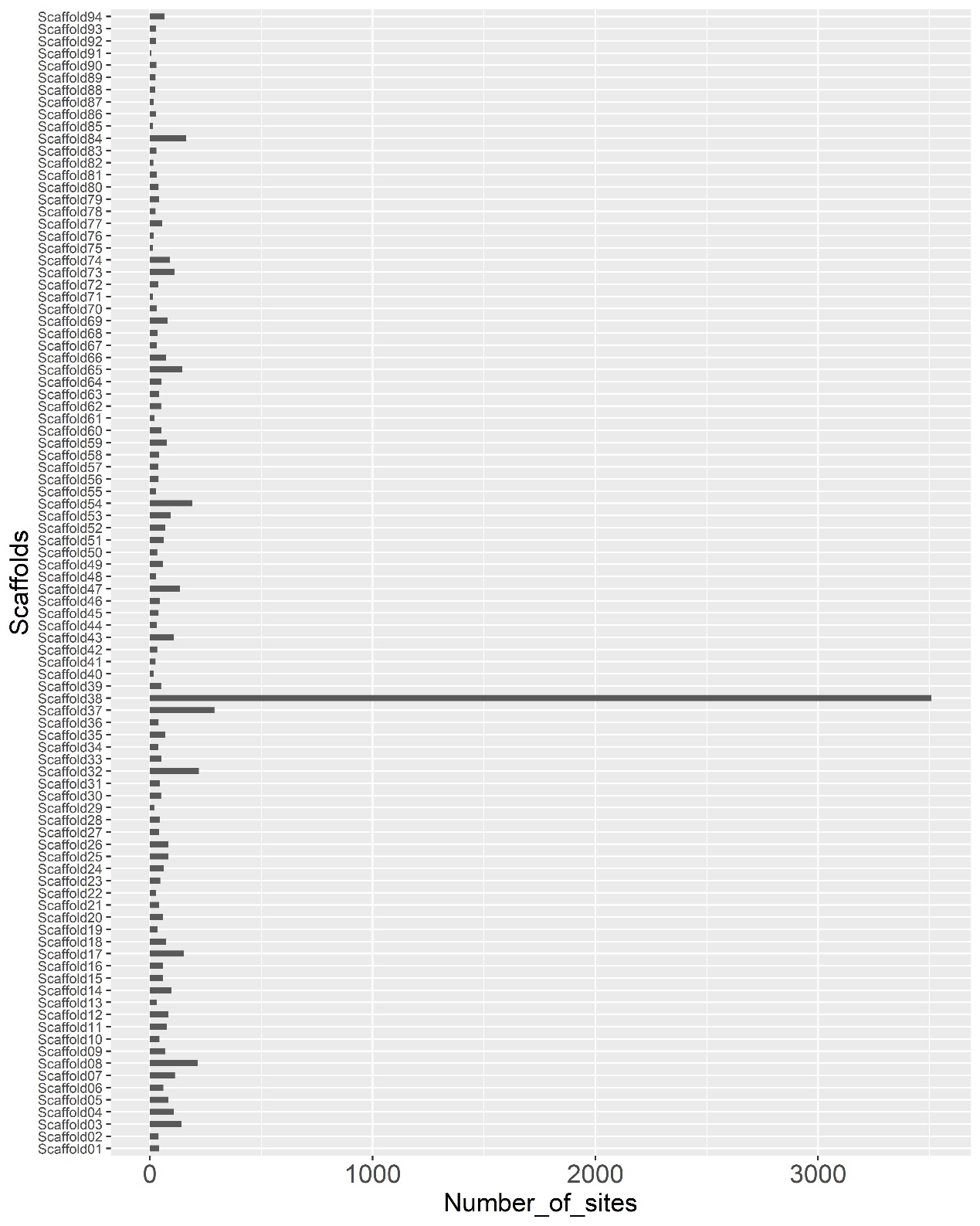


**Supplementary Figure S2** Statistics of SNP and indel molecular markers on all scaffolds. The horizontal axis is number of sites. The vertical ordinate is scaffolds.
