# Supplementary material for: Exploration of an XX/XY Sex Determination System and Development of PCR-Based Sex-specific Markers in Procambarus clarkii Based on Next-Generation Sequencing Data
Source: Front Genet. 2022 Mar 1;13:850983. doi: 10.3389/fgene.2022.850983 (PMC8923706; doi:10.3389/fgene.2022.850983)
Supplement: Supplementary file 1 [file DataSheet1.ZIP › Supplemental materials/Table S1.docx]

**Supplementary Table S1.** The sample sites and the number of individuals

| Location | Chaohu | Gaoyou | Yangxin | Honghu | Hanchuan | Total |
| --- | --- | --- | --- | --- | --- | --- |
| Number of females | 20 | 15 | 28 | 22 | 20 | 105 |
| Number of males | 21 | 25 | 8 | 17 | 20 | 91 |
| Total | 41 | 40 | 36 | 39 | 40 | 196 |
