# Supplementary figures and images for: Exploration of an XX/XY Sex Determination System and Development of PCR-Based Sex-specific Markers in Procambarus clarkii Based on Next-Generation Sequencing Data
Source: Front Genet. 2022 Mar 1;13:850983. doi: 10.3389/fgene.2022.850983 (PMC8923706; doi:10.3389/fgene.2022.850983)

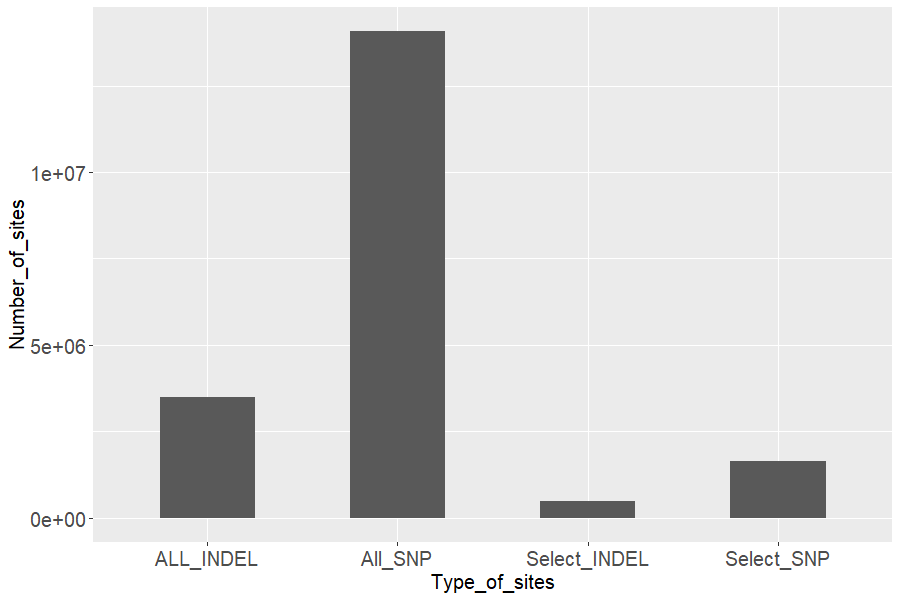


**Supplementary Figure S1** The distribution of SNP and InDel sites obtained.

Supplement: Supplementary file 1 [file DataSheet1.ZIP › Supplemental materials/Figure S1.docx]
